# Analysis of the impact of parents' electronic screen time habits, young children's screen exposure and parent-child interaction on language development delay in young children

**DOI:** 10.3389/fped.2025.1667048

**Published:** 2025-08-28

**Authors:** Xiaohong Wan, Xiaoqing Kang, Shunli Chen, Juan Du, Fang Yan, Yongqi Bai

**Affiliations:** ^1^ Department of Child Health Care, Children’s Medical Center, The Affiliated Hospital of Southwest Medical University, Sichuan Clinical Research Center for Birth Defects, Luzhou, Sichuan, China; ^2^ Department of Pediatric Hematologic Oncology and Respiratory, Children’s Medical Center, The Affiliated Hospital of Southwest Medical University, Sichuan Clinical Research Center for Birth Defects, Luzhou, Sichuan, China

**Keywords:** language development delay, screen time, parent-child interaction, emotion regulation, nursing child assessment teaching scale

## Abstract

**Purpose:**

Language development delay (LDD) affected 5%–10% of preschool children globally, and modifiable environmental factors such as screen exposure drew significant attention. This study aimed to evaluate how parental screen habits, children's screen exposure, and parent-child interactions collectively influenced the risk of LDD.

**Methods:**

This study employed a retrospective case-control design involving young children who received health check-ups between October 2020 and October 2024. Participants were categorized into normal and Language Developmental Delay groups by Bayley Scales of Infant and Toddler Development-III (BSID-III). Parent and child screen time (ST) were measured using a 7-day diary. Parent-child interaction quality was assessed using the Nursing Child Assessment Teaching Scale (NCATS). Emotion regulation and parenting stress were evaluated using the Preschool Children's Emotional Regulation Strategies Questionnaire and the Parenting Stress Index.

**Results:**

Among 296 children (113 with LDD, 106 normal), parents in the LDD group spent significantly more time on screens daily (fathers: + 0.34 h; mothers: + 0.32 h) and had higher total entertainment time (+12.07 min). Children with LDD showed longer average daily screen exposure (+0.4 h), with 30.97% exceeding 2 h per day compared to 12.26% in the control group. The LDD group had lower parent-child interaction frequency (>3 times/week: 16.81% vs. 30.19%), lower storytelling rates (13.27% vs. 31.13%), and lower NCATS scores (96.52 vs. 99.45).

**Conclusion:**

This study emphasized the importance of modifiable environmental factors, particularly excessive parental and child ST and decreased interaction quality, in the risk of LDD. It highlighted the necessity for behavioral interventions at the family level.

## 1 Introduction

LDD represented a common neurodevelopmental disorder typically identified during early childhood (1). Clinically, children exhibited significant delays in expressive and receptive language skills (2). This condition affected approximately 5%–10% of preschool-aged children worldwide. Children often had limited vocabulary, simple sentence structures, and faced difficulties in social interactions, which could lead to academic and psychosocial challenges (3). While genetic and neurobiological factors influenced the onset of LDD, growing research highlighted the crucial role of modifiable environmental factors (4, 5). Speech and language therapy served as the primary intervention method, but its effectiveness frequently suffered from late diagnosis and varied treatment responses among different children (6). With the rapid proliferation of digital devices, concerns increased regarding the impact of electronic screen exposure on children (7). However, the relationship between parental screen use habits, children's screen exposure, and parent-child interaction remained underexplored. Our study aimed to address this gap by evaluating the collective impact of these factors on LDD and identifying more effective intervention strategies.

Increasing evidence indicated that excessive ST could disrupt language learning by affecting neurocognitive and sensory mechanisms (8, 9). Notably, parents' screen use potentially impacted children's development indirectly by reducing timely responses to their needs. Excessive ST impaired phonetic discrimination and reduced the quality of language input, thereby hindering language development (10). According to the displacement hypothesis, time spent on screens replaced crucial verbal exchanges and joint attention activities essential for child development (11, 12). The study showed that parents' device use disrupted the continuity of attention during caregiving activities (13). NCATS framework suggested that parent-child interactions build language skills through contingent responses, where caregivers interpreted and responded to children's cues, fostering neural pathways for communication (14). However, screen exposure disrupted this interaction pattern. When parents became distracted by devices, the “serve and return” exchanges with their children decreased, and children's own interactions with screens limited opportunities for conversational practice. Neuroimaging studies found that screen-based stimuli activated different brain regions compared to face-to-face social interactions, which might weaken the brain's response to human language (15). Additionally, background screen noise lowered the quality of language input and impaired phonetic discrimination, critical for early language development (16). These findings supported the hypothesis, suggesting that ST replaced key interactions necessary for language maturation (17). Excessive ST not only affected children's direct language learning opportunities but also indirectly hindered their language development by reducing high-quality parent-child interactions.

This retrospective case-control study explored how parental screen use habits, children's screen exposure time, and the quality of parent-child interactions collectively influenced the risk of LDD in young children. To better understand the relationships among these factors, researchers conducted a multidimensional analysis of family digital behaviors and interaction patterns. The study aimed to determine the individual and combined effects of parental screen habits, child screen exposure, and deficiencies in parent-child interaction on LDD risk. It also sought to identify modifiable protective factors and integrate key predictors into a clinically applicable risk assessment tool. The innovation of this study lay in simultaneously evaluating screen dynamics for both parents and children and developing a predictive tool for early risk stratification. By identifying modifiable family digital behaviors, this research provided practical strategies for preventing and promptly intervening in pediatric language disorders.

## 2 Materials and methods

### 2.1 Research design

This is a retrospective case-control study aimed at evaluating the impact of parental ST habits, screen exposure in young children, and parent-child interaction on LDD in young children. The participants were recruited through the hospital's routine health check-up programs, which include regular physical examinations for children from newborn to preschool age, covering basic physical examinations and also including assessments of language development and other key areas. Participants’ ages were recorded at the time of recruitment. The study included 296 young children who underwent health check-ups at our hospital between October 2020 and October 2024. The comprehensive neurodevelopmental assessment included the receptive and expressive language sub-scales of BSID-III. The raw scores of each item were converted to scaled scores, with a score <7 being diagnosed as LDD (18). It was clarified that the BSID-III would be used as a diagnostic tool, emphasizing the integration of clinical judgment from professional speech therapists to ensure the accuracy of the diagnosis. Based on whether they were diagnosed with language delay, 219 children were divided into two groups: the Language Developmental Delay Group and the Normal Group. One hundred thirteen children with LDD were included in the Language Developmental Delay Group, while 106 children with normal language development were included in the Normal Group.

To ensure the generalization ability and reliability of the model, we selected 77 toddlers who met the same inclusion criteria as an external validation set. Another external validation cohort consisted of 77 toddlers who met the same inclusion criteria but were evaluated separately to validate the predictive model. During the same period, this validation group was collected under the same procedures as the primary cohort to ensure consistency in data collection methods. Depending on whether they were diagnosed with LDD, these children were also categorized into the Language Developmental Delay Group (*n* = 41) and the Normal Group (*n* = 36). The validation cohort was not part of the initial 296 participants, representing an independent sample used for external validation purposes.

### 2.2 Ethical approval

All procedures conducted in this study were in accordance with the ethical standards of the institutional and/or national research committee and with the 1964 Helsinki Declaration and its later amendments. Informed consent was obtained from all caregivers prior to participation. Written informed consent was obtained for the use of video recordings and observational data in interaction-based assessments. Caregivers were assured that all data would be anonymized and treated confidentially. Confidentiality was ensured by assigning a unique identifier to each participant and removing any personally identifiable information from video recordings and observation notes before coding. Additionally, all observational coding was performed by trained researchers who were unaware of the participants' identities or group assignments.

### 2.3 Inclusion and exclusion criteria

The inclusion criteria are as follows: (1) Children aged 12–48 months; (2) The child's parents are over 18 years old; (3) At least one parent uses smartphones, tablets, or other electronic devices daily; (4) Complete medical records and follow-up data are available.

The exclusion criteria are as follows: (1) Premature birth (gestational age <37 weeks) or low birth weight (<2,500 g); (2) The toddler has been diagnosed with Autism Spectrum Disorder (ASD), intellectual disability, or other neurodevelopmental disorders that may significantly affect language development; (3) The toddler suffers from severe chronic illness or is undergoing treatment; (4) The toddler has known hearing impairment or visual impairment; (5) There is severe domestic violence or an unstable family environment in the household.

### 2.4 Screen exposure

Screen exposure of parents and children was assessed using the Child and Family Experiences (CAFE) tool. The CAFE tool is a comprehensive assessment framework that integrates multiple methods to evaluate household media exposure, including internet-based surveys, screen use diaries, and passive sensing applications installed on family mobile devices. Passive sensing applications were specifically developed for Android devices (version 8.0 and above) and iOS devices (version 13.0 and above). These applications monitor various metrics related to device usage, including: total screen time—the total duration of device usage; app-specific screen time—the duration spent on specific applications. Usage frequency—the number of times the device or a specific application is accessed. Data is collected in 5-min intervals, allowing for detailed analysis of usage patterns throughout the day. Compliance is defined as having valid screen time data (i.e., recorded total screen time exceeding 8 h) for at least 6 out of 7 days. These applications run in the background without interfering with normal device operations, ensuring minimal impact on participants' daily lives while capturing comprehensive data on screen time and usage patterns (19).

Parental Screen Time Measurement: Parental screen time measurement is an integral component of the CAFE tool. Parents were provided with diary templates to log their own screen time and habits within the 7 days prior to diagnosis. These diaries were collected during follow-up periods. A health inspector contacted parents via phone to ensure accurate recording of their typical weekday and weekend screen use habits. Daily screen time data for parents, covering both recreational and social screen use, were collected over this 7-day observation period as part of the CAFE tool.

Children's Screen Time Measurement: Similarly, children's screen time measurement is also an integral part of the CAFE tool. Parents observed and recorded their children's screen time within the 7 days prior to diagnosis using diaries, which were later collected during follow-ups. A health inspector followed up with parents by phone to ensure accurate daily recording of children's screen time. For children attending school or daycare, teachers documented the child's screen time as requested through letters sent by the parents. The average screen time per day over the 7-day observation period was considered as the child's screen time, and additional details such as age of first screen exposure and content viewed were also collected.

### 2.5 Parent-child interaction

To develop a questionnaire in combination with the United Nations Children's Fund (UNICEF) Multiple Indicator Cluster Survey (MICS) (20), aimed at collecting information on parent-child interactions of children within the 3 days prior to diagnosis. The forms of parent-child interaction include reading or looking at picture books with the child, telling stories to the child, singing songs to the child or singing together, taking the child out, playing with the child, and engaging in activities such as identifying objects, counting, or drawing with the child.

NCATS includes 73 parent and child behaviors that are observed and recorded as “observed” or “not observed” during teaching interactions. The predictor variable scores used in the analysis include both parent scores and child scores. Parent scores are the sum of four subscales (coping with distress, fostering socioemotional growth, fostering cognitive growth, and sensitivity to cues); child scores are the sum of two subscales (clarity of cues and responsiveness to caregiver). Each indicator is given a score of 0, 1, or 2 based on the quality of the interaction, with a total possible score ranging from 0 to 146, where higher scores indicate better interaction outcomes. The Cronbach's alpha for NCATS was reported as 0.74 (21).

### 2.6 Parenting stress and emotion regulation strategies questionnaire

Parenting Stress Questionnaire: This study adopted the Abidin Parenting Stress Index prior to diagnosis. The questionnaire consists of 36 items divided into three dimensions: dysfunctional parent-child interactions, parenting stress, and difficult child. Each dimension comprises 12 items. The questionnaire uses a Likert 5-point scale ranging from 1 (strongly disagree) to 5 (strongly agree), with higher scores indicating greater parenting stress. The internal consistency coefficient (Cronbach's *α*) for each dimension is 0.827 (22).

Emotion Regulation Strategies Questionnaire: This study utilized the Preschool Children's Emotional Regulation Strategies Questionnaire prior to diagnosis. The questionnaire consists of 48 items divided into eight dimensions. Positive emotional regulation strategies include five dimensions, while negative emotional regulation strategies encompass three dimensions. The three dimensions of negative emotional regulation strategies selected are: passive coping (6 items), emotional outbursts (7 items), and aggressive behavior (4 items). The scale uses a 5-point Likert scale ranging from 1 (never) to 5 (always), with separate scores for the three dimensions. Higher scores indicate a greater ability of the child to use emotional regulation strategies. The internal consistency coefficient for this questionnaire (Cronbach's *α*) is 0.75 (23).

### 2.7 Statistical method

Data collection and analysis were both conducted by the same individual. Data analysis was performed using SPSS 29.0 statistical software (SPSS Inc., Chicago, IL, USA). Categorical data are presented in the format of [*n* (%)]. Chi-square tests were used when sample size was ≥40 and theoretical frequency (T) ≥5, with the test statistic denoted as chi-square (*χ*^2^). If the sample size was ≥40 but the theoretical frequency fell within the range of 1 ≤ T < 5, a correction formula was applied to adjust the chi-square test. For continuous data following a normal distribution, results are expressed as (mean ± standard deviation), and *t*-tests were used for comparisons. Pearson correlation analysis was conducted for continuous variables, while Spearman correlation analysis was used for categorical variables. Univariate and multivariate logistic regression analyses were performed. Univariate logistic regression analysis was utilized to evaluate the independent effects of parental electronic ST habits, young children's screen exposure, and parent-child interaction on delayed language development in children. Variables showing statistical significance in univariate analysis were subsequently included in the multivariate logistic regression model to adjust for potential confounding factors and identify independent predictors of delayed language development in children. The results of logistic regression analysis are reported as odds ratios (ORs) with corresponding 95% confidence intervals (Cis) and *p*-values. A *p*-value <0.05 was considered statistically significant. This study has no missing values.

## 3 Results

### 3.1 Analysis of differences in general information of two groups

Children's age (*t* = 0.101, *p* = 0.919), parents' mean age (*t* = 0.381, *p* = 0.704), gestational age at birth (*t* = 0.534, *p* = 0.594), and birth weight (*t* = 0.380, *p* = 0.704) did not show significant differences between the two groups (Table 1). Similarly, gender distribution (*χ*^2^ = 0.740, *p* = 0.390), mode of delivery (*χ*^2^ = 1.321, *p* = 0.250), caregiver identity (*χ*^2^ = 0.044, *p* = 0.834), parents' ethnicity (*χ*^2^ = 0.054, *p* = 0.816), and education level of the caregiver (*χ*^2^ = 1.276, *p* = 0.735) also exhibited no significant differences. Household income approached significance but did not meet the threshold for statistical significance (*χ*^2^ = 5.136, *p* = 0.077). These results indicate that most general characteristics, including demographic and socioeconomic factors, do not significantly differ between the Normal Group and the Language Development Delay Group. This suggests that these variables may not be primary contributors to developmental delays in this cohort.

**Table 1:** Comparison of general information between two groups.

Parameters	Normal group (*n* = 106)	Language developmental delay group (*n* = 113)	*t*/*χ*^2^	*p*
Children age (months)	27.74 ± 8.65	27.86 ± 7.44	0.101	0.919
Parents mean age (years)	29.34 ± 3.56	29.52 ± 3.41	0.381	0.704
Gender [*n* (%)]			0.740	0.390
Male	52 (49.06%)	62 (54.87%)		
Female	54 (50.94%)	51 (45.13%)		
Gestational age at birth (weeks)	40.11 ± 1.45	40.02 ± 1.13	0.534	0.594
Birth weight (kg)	3.42 ± 0.14	3.41 ± 0.21	0.380	0.704
Mode of delivery [*n* (%)]			1.321	0.250
Natural birth	69 (65.09%)	65 (57.52%)		
Cesarean section	37 (34.91%)	48 (42.48%)		
Caregiver [*n* (%)]			0.044	0.834
Mother	88 (83.02%)	95 (84.07%)		
Father	18 (16.98%)	18 (15.93%)		
Parents ethnics [*n* (%)]			0.054	0.816
Han	83 (78.30%)	87 (76.99%)		
Minority	23 (21.70%)	26 (23.01%)		
Education of Caregiver [*n* (%)]			1.276	0.735
High school and above	14 (13.21%)	13 (11.50%)		
Junior high	37 (34.91%)	36 (31.86%)		
Primary	41 (38.68%)	43 (38.05%)		
None	14 (13.21%)	21 (18.58%)		
Household income [*n* (%)]			5.136	0.077
<5,000 RMB/month	4 (3.77%)	13 (11.50%)		
5,000–9,999 RMB/month	76 (71.70%)	79 (69.91%)		
>9,999 RMB/month	26 (24.53%)	21 (18.58%)		

### 3.2 Comparison of parents' electronic ST habits between two groups

Father's daily ST showed a significant difference (*t* = 3.154, *p* = 0.002) between the two groups, with fathers in the Language Developmental Delay Group spending more time on screens compared to those in the Normal Group (Table 2). Similarly, mother's daily ST also exhibited a significant difference (*t* = 2.624, *p* = 0.009), indicating higher ST among mothers in the Language Developmental Delay Group. For entertainment usage, weekday entertainment (*t* = 1.275, *p* = 0.204), weekend entertainment (*t* = 1.368, *p* = 0.173), and total entertainment time (*t* = 2.579, *p* = 0.011) were examined. Only total entertainment time demonstrated a significant difference, with parents in the Language Developmental Delay Group spending more time on entertainment activities. Regarding social networking, no significant differences were observed for weekday social networking (*t* = 0.584, *p* = 0.560), weekend social networking (*t* = 1.215, *p* = 0.226), or total social networking time (*t* = 0.882, *p* = 0.379).

**Table 2:** Comparison of parents’ electronic screen time habits between two groups.

Parameters	Normal group (*n* = 106)	Language developmental delay group (*n* = 113)	*t*	*p*
Father's daily screen time (hours)	2.03 ± 0.61	2.37 ± 0.95	3.154	0.002
Mother's daily screen time (hours)	2.08 ± 0.69	2.40 ± 1.07	2.624	0.009
Weekday entertainment (min)	97.42 ± 12.75	101.68 ± 32.99	1.275	0.204
Weekend entertainment (min)	130.45 ± 32.45	136.56 ± 33.56	1.368	0.173
Total entertainment (min)	227.56 ± 33.56	239.63 ± 35.56	2.579	0.011
Weekday social networking (min)	30.63 ± 11.45	31.46 ± 9.35	0.584	0.560
Weekend social networking (min)	34.57 ± 6.56	36.57 ± 16.13	1.215	0.226
Total social networking (min)	64.65 ± 12.45	66.06 ± 11.26	0.882	0.379

### 3.3 Comparison of young children's screen exposure between two groups

Age at first screen exposure did not show a significant difference between the two groups (*t* = 0.760, *p* = 0.449) (Table 3). However, average daily screen exposure time was significantly different (*t* = 2.425, *p* = 0.016), with children in the Language Developmental Delay Group having higher exposure times. Stratification of average daily screen exposure time also revealed a significant difference (*χ*^2^ = 11.204, *p* = 0.004), indicating that a greater proportion of children in the Language Developmental Delay Group were exposed to more than 2 h of ST per day compared to the Normal Group. For the content of screen viewing, no significant differences were observed for cartoons (*χ*^2^ = 3.375, *p* = 0.066), educational and intellectual content (*χ*^2^ = 2.620, *p* = 0.106), or entertainment content (*χ*^2^ = 0.018, *p* = 0.892). These results suggest that higher daily screen exposure time is associated with developmental delays.

**Table 3:** Comparison of young children's screen exposure between two groups.

Parameters	Normal group (*n* = 106)	Language developmental delay group (*n* = 113)	*t*/χ^2^	*p*
Age at first screen exposure (months)	16.74 ± 1.65	16.86 ± 0.44	0.760	0.449
Average daily screen exposure time (hours)	2.86 ± 1.05	3.26 ± 1.37	2.425	0.016
Stratification of average daily screen exposure time [*n* (%)]			11.204	0.004
<1 h/d	51 (48.11%)	42 (37.17%)		
1–2 h/d	42 (39.62%)	36 (31.86%)		
>2 h/d	13 (12.26%)	35 (30.97%)		
Content of screen viewing [*n* (%)]				
Cartoons	83 (78.30%)	99 (87.61%)	3.375	0.066
Educational and intellectual content	12 (11.32%)	6 (5.31%)	2.620	0.106
Entertainment content	45 (42.45%)	49 (43.36%)	0.018	0.892

### 3.4 Comparison of parent-child interaction between two groups

Parent-child interaction frequency showed a significant difference (*χ*^2^ = 5.477, *p* = 0.019) between the two groups, with fewer parents in the Language Developmental Delay Group reporting more than three interactions per week compared to the Normal Group (Table 4). Telling stories exhibited a significant difference (*χ*^2^ = 10.192, *p* = 0.001), with a notably lower percentage of parents in the Language Developmental Delay Group engaging in this activity compared to those in the Normal Group. Reading books (*χ*^2^ = 1.101, *p* = 0.294), singing (*χ*^2^ = 0.181, *p* = 0.671), taking children outdoors (*χ*^2^ = 0.054, *p* = 0.816), playing together (*χ*^2^ = 1.216, *p* = 0.270), and recognizing things (*χ*^2^ = 1.316, *p* = 0.251) did not show significant differences between the groups. The NCATS score indicated a significant difference (*t* = 2.404, *p* = 0.017), suggesting that children in the Normal Group had higher scores compared to those in the Language Developmental Delay Group. These findings highlight that the frequency of parent-child interactions and storytelling are significantly associated with developmental outcomes.

**Table 4:** Comparison of parent-child interaction between two groups.

Parameters	Normal group (*n* = 106)	Language developmental delay group (*n* = 113)	*t*/χ^2^	*p*
Parent-child interaction [*n* (%)]			5.477	0.019
≤3 times	24 (30.19%)	19 (16.81%)		
≤3 times	74 (69.81%)	94 (83.19%)		
Reading books [*n* (%)]	32 (30.19%)	27 (23.89%)	1.101	0.294
Telling stories [*n* (%)]	27 (31.13%)	15 (13.27%)	10.192	0.001
Singing [*n* (%)]	27 (25.47%)	26 (23.01%)	0.181	0.671
Taking outdoors [*n* (%)]	83 (78.30%)	87 (76.99%)	0.054	0.816
Playing together [*n* (%)]	51 (48.11%)	46 (40.71%)	1.216	0.270
Recognizing things [*n* (%)]	57 (53.77%)	52 (46.02%)	1.316	0.251
NCATS score	99.45 ± 8.23	96.52 ± 9.67	2.404	0.017

TNF-α, tumor necrosis factor-alpha; IL, interleukin; sFas, soluble fas.

### 3.5 Comparison of parenting stress and emotion regulation strategies between two groups

**Figure 1:**
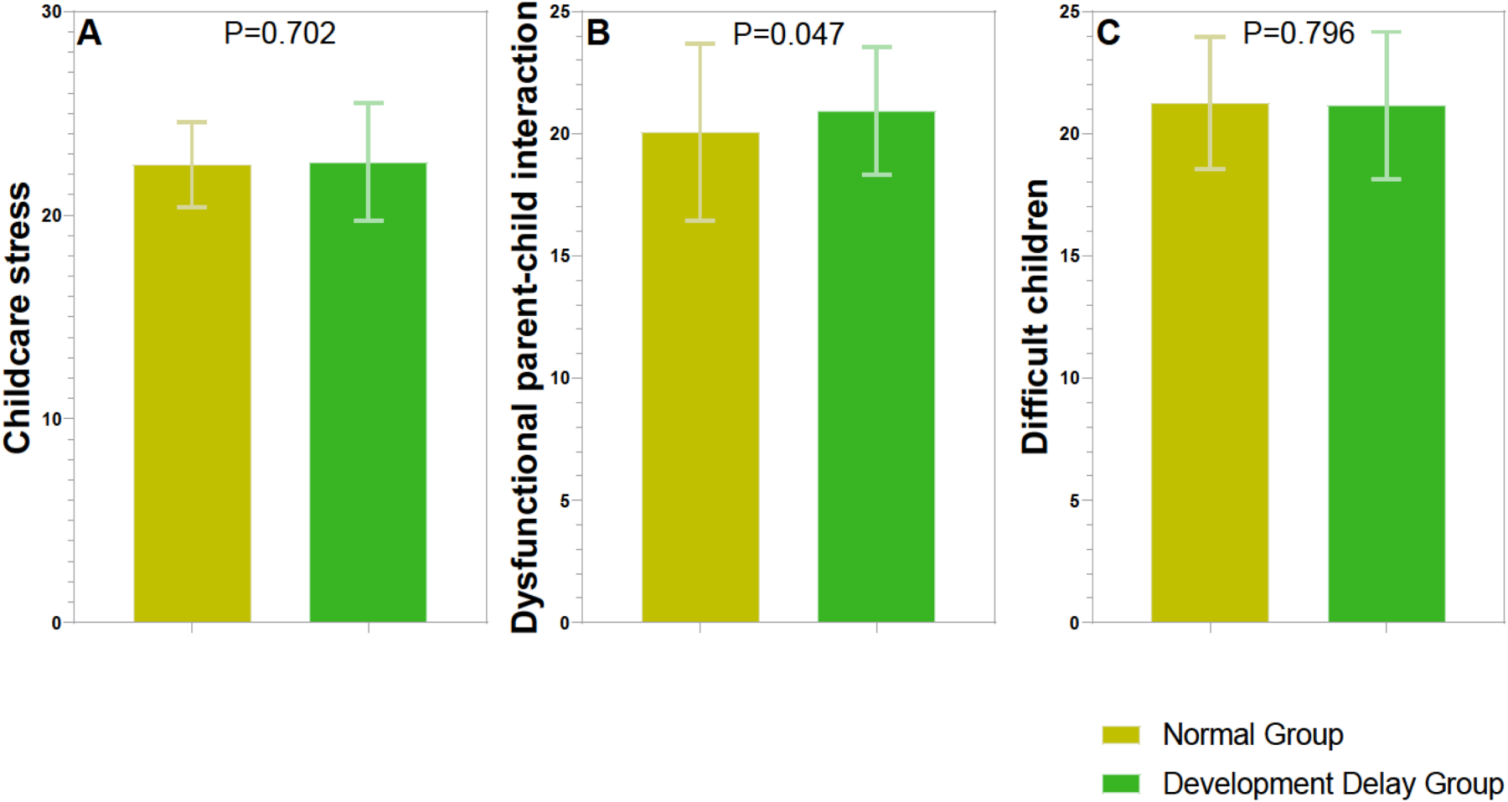
Comparison of parenting stress index between two groups. **(A)** Childcare stress (score); **(B)** dysfunctional parent-child interaction (score); **(C)**. Difficult children (score).

Childcare stress did not show a significant difference between the two groups (*t* = 0.383, *p* = 0.702) (Figure 1). However, dysfunctional parent-child interaction scores indicated a significant difference (*t* = 2.001, *p* = 0.047), with higher scores observed in the Language Developmental Delay Group compared to the Normal Group. Difficult children scores also did not show a significant difference (*t* = 0.259, *p* = 0.796) between the groups. These results suggest that while general childcare stress and perceptions of children's difficulty levels do not differ significantly between parents of children with normal development and those with developmental delays, the quality of parent-child interactions may be more strained in families with children experiencing developmental delays.

**Figure 2:**
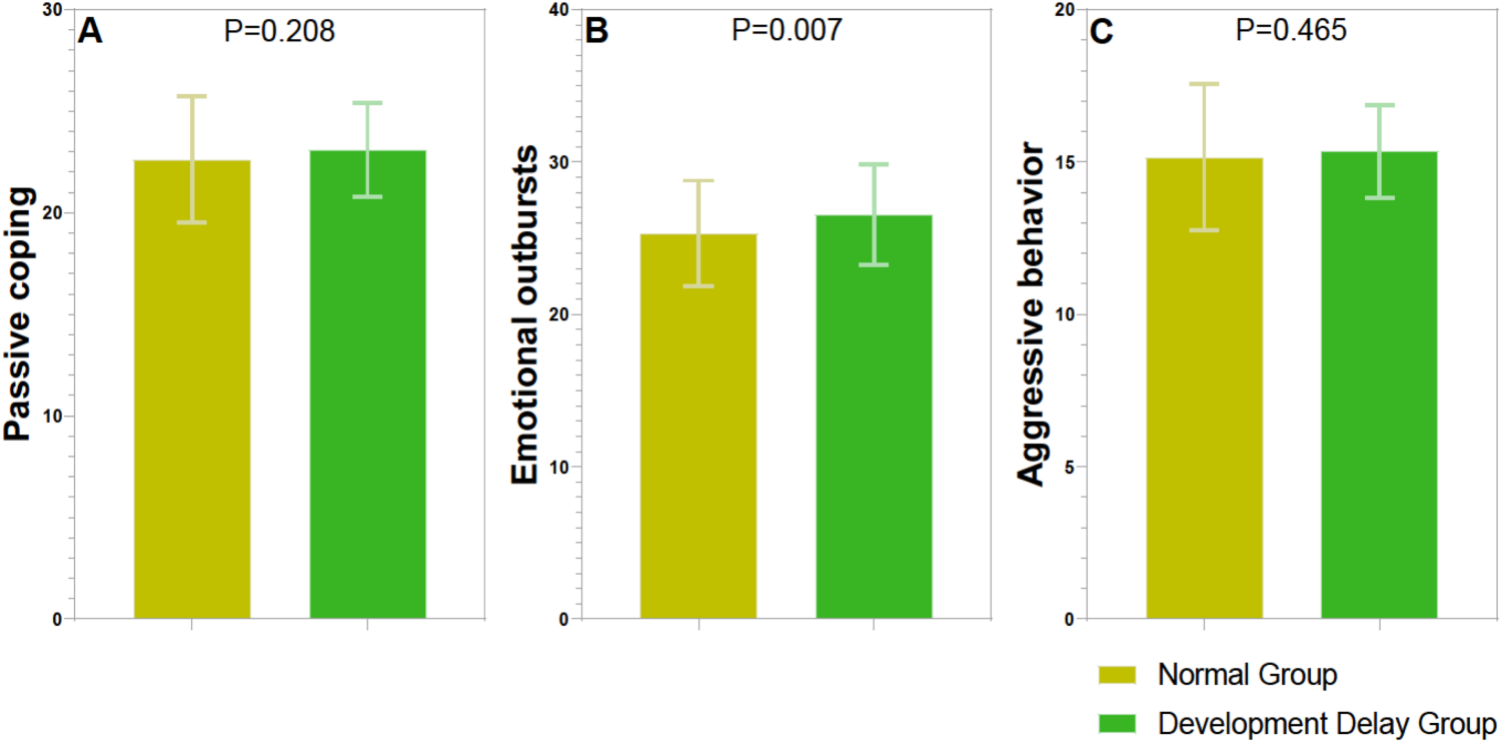
Comparison of emotion regulation strategies questionnaire between two groups. **(A)** Passive coping (score); **(B)** emotional outbursts (score); **(C)** aggressive behavior (score).

For emotion regulation strategies, passive coping did not show a significant difference between the two groups (*t* = 1.263, *p* = 0.208) (Figure 2). However, emotional outbursts were significantly different (*t* = 2.714, *p* = 0.007), with higher scores observed in the Language Developmental Delay Group compared to the Normal Group. Aggressive behavior also did not show a significant difference (*t* = 0.732, *p* = 0.465) between the groups. This suggests that children with developmental delays may exhibit more frequent or intense emotional outbursts.

### 3.6 Correlation analysis

In the correlation analysis examining the relationship between various variables and language development delay in young children, several factors showed statistically significant associations (*P* < 0.05) (Table 5). Father's daily screen time (*P* = 0.004), mother's daily screen time (*P* = 0.007), total entertainment (*P* = 0.008), average daily screen exposure time (*P* = 0.020), stratification of average daily screen exposure time (*P* = 0.008), and emotional outbursts (*P* = 0.017) were significantly positively correlated with the occurrence of language developmental delay. On the other hand, parent-child interaction (*P* = 0.019), telling stories (*P* = 0.001), and NCATS score (*P* = 0.022) were significantly negatively correlated with the occurrence of language developmental delay. Dysfunctional parent-child interaction did not show a significant association with language development delay (*P* = 0.063).

**Table 5:** Correlation analysis between father's daily screen time, mother's daily screen time, total entertainment, average daily screen exposure time, stratification of average daily screen exposure time, parent-child interaction, telling stories, NCATS score, dysfunctional parent-child interaction and emotional outbursts and language development delay in young children.

Variable	rho	*p*
Father's daily screen time	0.195	0.004
Mother's daily screen time	0.181	0.007
Total entertainment	0.180	0.008
Average daily screen exposure time	0.158	0.020
Stratification of average daily screen exposure time	0.179	0.008
Parent-child interaction	−0.158	0.019
Telling stories	−0.216	0.001
NCATS score	−0.155	0.022
Dysfunctional parent-child interaction	0.126	0.063
Emotional outbursts	0.161	0.017

### 3.7 Regression analysis of language development delay in young children

In the regression analysis of factors affecting language development delay in young children, father's daily ST and mother's daily ST were significantly associated with increased risk in both univariate (*p* = 0.003 and *p* = 0.011, respectively) and multivariate analyses (*p* = 0.006 and *p* = 0.024, respectively) (Table 6). Total entertainment time showed significant associations in both univariate (*p* = 0.012) and multivariate analyses (*p* = 0.036). Stratification of average daily screen exposure time was significant in both univariate (*p* = 0.005) and multivariate analyses (*p* = 0.003). Telling stories had a significant protective effect in both univariate (*p* = 0.002) and multivariate analyses (*p* = 0.041), indicating its potential role in mitigating language development delays. The NCATS score was significantly protective in both univariate (*p* = 0.019) and multivariate analyses (*p* = 0.022), further supporting the importance of certain parental behaviors and child assessments. Emotional outbursts were significantly associated with increased risk in both univariate (*p* = 0.008) and multivariate analyses (*p* = 0.036), indicating an increased risk of language development delay. Parent-child interaction frequency showed a trend towards significance in univariate analysis (*p* = 0.021) but did not reach significance in multivariate analysis (*p* = 0.117), suggesting it may still play a role in mitigating developmental risks.

**Table 6:** Univariate and multivariate regression analysis of the impact of father's daily screen time, mother's daily screen time, total entertainment, average daily screen exposure time, stratification of average daily screen exposure time, parent-child interaction, telling stories, NCATS score, dysfunctional parent-child interaction and emotional outbursts on language development delay in young children.

Parameters	Univariate analysis			Multivariate analysis		
	*P*	OR	95%Cl	*P*	OR	95%CI
Father's daily screen time	0.003	1.710	1.214–2.462	0.006	1.763	1.178–2.638
Mother's daily screen time	0.011	1.476	1.097–2.010	0.024	1.501	1.054–2.139
Total entertainment	0.012	1.010	1.002–1.019	0.036	1.010	1.001–1.019
Average daily screen exposure time	0.018	1.306	1.050–1.639	0.073	1.268	0.978–1.645
Stratification of average daily screen exposure time	0.005	1.657	1.168–2.376	0.003	1.888	1.239–2.878
Parent-child interaction	0.021	0.467	0.242–0.883	0.117	0.538	0.248–1.168
Telling stories	0.002	0.339	0.167–0.659	0.041	0.451	0.211–0.966
NCATS score	0.019	0.964	0.935–0.993	0.022	0.960	0.927–0.994
Dysfunctional parent-child interaction	0.047	1.092	1.003–1.194	0.069	1.099	0.993–1.217
Emotional outbursts	0.008	1.115	1.030–1.212	0.036	1.106	1.006–1.216

### 3.8 ROC curve analysis of parents' electronic ST habits, young children's screen exposure and parent-child interaction on language development delay in young children

The ROC curve analysis indicated that father's and mother's daily ST, along with total entertainment time, showed moderate discriminatory ability for predicting language development delays in young children (Table 7). These factors had AUC values around 0.6, suggesting reasonable predictive power. Emotional outbursts also demonstrated a moderate ability to predict language development delays. In contrast, parent-child interaction and telling stories had low AUC values, indicating poor discriminatory power for identifying children at risk of language development delays. The NCATS score provided moderate predictive ability but was less robust compared to parental ST and emotional outbursts.

**Table 7:** ROC curve analysis of father's daily screen time, mother's daily screen time, total entertainment, average daily screen exposure time, stratification of average daily screen exposure time, parent-child interaction, telling stories, NCATS score, dysfunctional parent-child interaction and emotional outbursts on language development delay in young children.

Parameters	Best threshold	Sensitivities	Specificities	AUC	Youden index	F1 score
Father's daily screen time	2.580	0.451	0.849	0.613	0.300	0.567
Mother's daily screen time	2.660	0.451	0.821	0.605	0.272	0.557
Total entertainment	230.940	0.611	0.594	0.604	0.205	0.613
Average daily screen exposure time	3.845	0.345	0.849	0.591	0.194	0.464
Stratification of average daily screen exposure time	1.500	0.310	0.877	0.597	0.187	0.435
Parent-child interaction	0.500	1.000	0.000	0.433	0.000	0.681
Telling stories	0.500	1.000	0.000	0.411	0.000	0.681
NCATS score	101.190	0.726	0.443	0.590	0.169	0.325
Dysfunctional parent-child interaction	17.775	0.894	0.245	0.573	0.139	0.687
Emotional outbursts	27.155	0.460	0.726	0.593	0.186	0.531

### 3.9 Development of a nomogram prediction model for parents' electronic ST habits, young children's screen exposure and parent-child interaction influencing language development delay in young children

**Figure 3:**
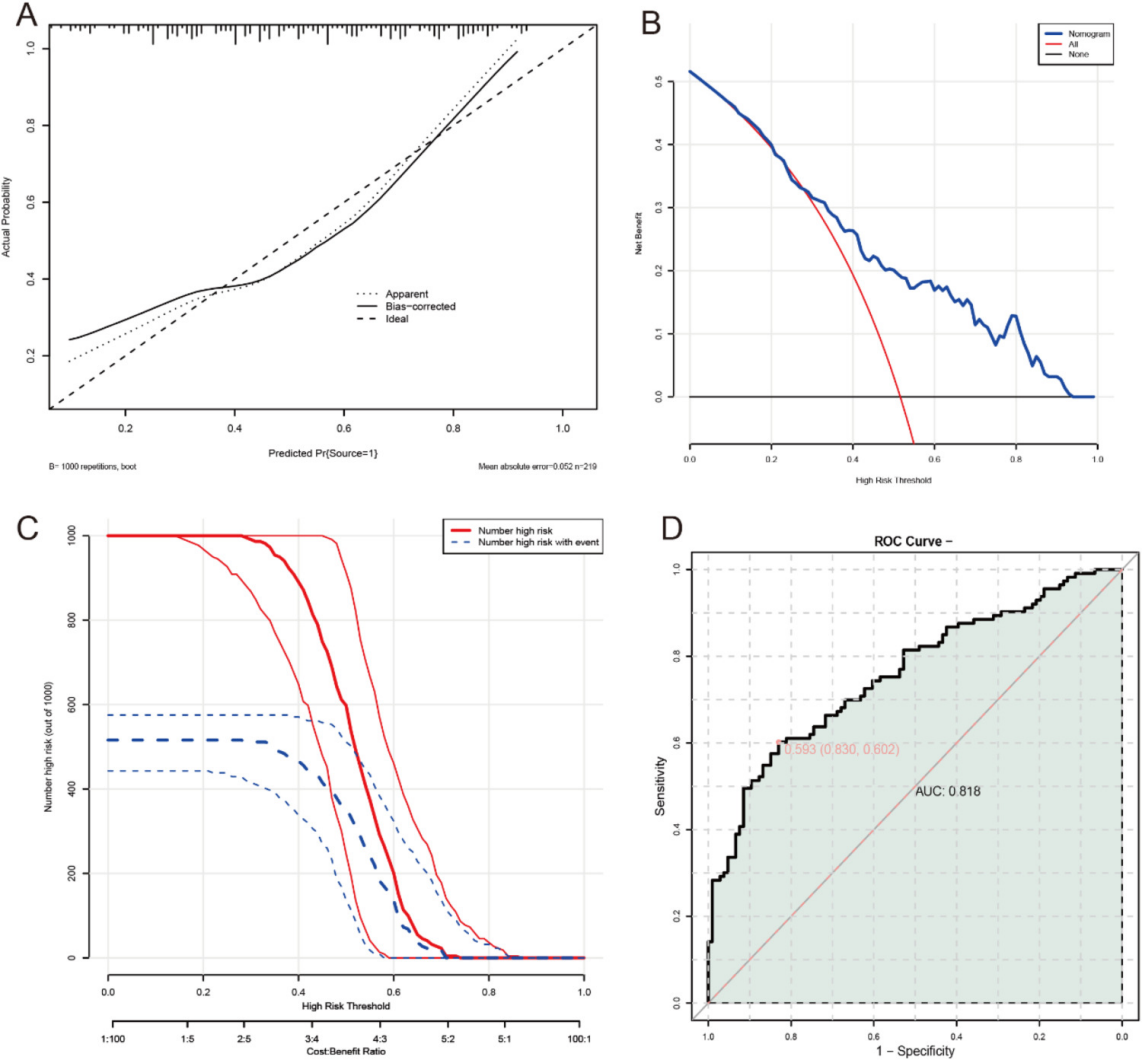
Development of a nomogram prediction model for father's daily screen time, mother's daily screen time, total entertainment, average daily screen exposure time, stratification of average daily screen exposure time, parent-child interaction, telling stories, NCATS score, dysfunctional parent-child interaction and emotional outbursts influencing language development delay in young children. **(A)** Calibration curve; **(B)** decision curve; **(C)** clinical impact curve; **(D)** ROC curve.

In the provided figure, our model demonstrates substantial predictive accuracy with an AUC of 0.818, indicating a significant ability to distinguish between individuals who will experience the event and those who will not (Figure 3). The calibration plot after correction for overfitting shows close agreement between predicted probabilities and actual outcomes, suggesting minimal bias in predictions. In terms of classification at various thresholds, there is a notable trade-off between the number of high-risk individuals identified and the number of true positive cases detected, with no statistically significant difference observed across different thresholds (*P* > 0.05). The decision curve analysis reveals that the model provides the greatest net benefit when the threshold probability ranges from 10% to 40%, compared to the strategies of treating all or none. Overall, these results indicate that our model performs well in identifying high-risk individuals within a specific range of probabilities, thereby supporting its application in clinical settings for improved risk assessment and patient management.

### 3.10 External validation of the predictive model

In the comparison of general information between the Normal Group and the Language Developmental Delay Group in the external test set, several parameters showed no significant differences (all *p* > 0.05), including children's age, parents' mean age, gender distribution, gestational age at birth, and birth weight, among others such as mode of delivery, primary caregiver, and household income (Table 8). However, significant differences were observed in other aspects. Fathers' daily ST (*p* = 0.007), mothers' daily ST (*p* = 0.027), total entertainment time (*p* = 0.032), average daily screen exposure time (*p* = 0.004), and stratification of average daily screen exposure time (*p* = 0.017) all indicated higher screen usage in the Language Developmental Delay Group. Additionally, parent-child interaction frequency (*p* = 0.009), telling stories (*p* = 0.002), dysfunctional parent-child interaction score (*p* = 0.008), and emotional outbursts score (*p* = 0.005) also showed significant differences, suggesting that reduced interaction and increased ST may correlate with developmental delays.

**Table 8:** Comparison of general information between two groups in the external test set.

Parameters	Normal group (*n* = 36)	Language developmental delay group (*n* = 41)	*t*/χ^2^	*p*
Children age (months)	27.73 ± 8.24	27.42 ± 7.47	0.169	0.866
Parents mean age (years)	29.25 ± 3.73	30.21 ± 3.31	1.191	0.237
Gender [*n* (%)]			0.540	0.463
Male	24 (66.67%)	24 (58.54%)		
Female	12 (33.33%)	17 (41.46%)		
Gestational age at birth (weeks)	40.13 ± 1.42	40.52 ± 1.74	1.079	0.284
Birth weight (kg)	3.31 ± 0.24	3.36 ± 0.32	0.763	0.448
Mode of delivery [*n* (%)]			0.002	0.966
Natural birth	23 (63.89%)	26 (63.41%)		
Cesarean section	13 (36.11%)	15 (36.59%)		
Caregiver [*n* (%)]			0.324	0.569
Mother	28 (77.78%)	34 (82.93%)		
Father	8 (22.22%)	7 (17.07%)		
Parents ethnics [*n* (%)]			2.089	0.148
Han	29 (80.56%)	27 (65.85%)		
Minority	7 (19.44%)	14 (34.15%)		
Education of parents [*n* (%)]			1.462	0.691
High school and above	4 (11.11%)	5 (12.20%)		
Junior high	14 (38.89%)	13 (31.71%)		
Primary	15 (41.67%)	16 (39.02%)		
None	3 (8.33%)	7 (17.07%)		
Household income [*n* (%)]			0.586	0.746
<5,000 RMB/month	2 (5.56%)	4 (9.76%)		
5,000–9,999 RMB/month	26 (72.22%)	27 (65.85%)		
>9,999 RMB/month	8 (22.22%)	10 (24.39%)		
Father's daily screen time (hours)	2.05 ± 0.67	2.54 ± 0.85	2.759	0.007
Mother's daily screen time (hours)	2.01 ± 0.57	2.46 ± 1.11	2.259	0.027
Weekday entertainment (min)	116.52 ± 12.11	123.21 ± 32.73	1.217	0.229
Weekend entertainment (min)	131.34 ± 32.67	136.31 ± 33.57	0.657	0.513
Total entertainment (min)	242.51 ± 33.11	259.65 ± 35.18	2.191	0.032
Weekday social networking (min)	30.31 ± 11.41	33.68 ± 9.26	1.432	0.156
Weekend social networking (min)	39.41 ± 6.31	41.55 ± 16.86	0.754	0.454
Total social networking (min)	64.06 ± 12.64	64.68 ± 11.74	0.222	0.825
Age at first screen exposure (months)	15.46 ± 1.73	15.53 ± 0.57	0.211	0.834
Average daily screen exposure time (hours)	2.61 ± 1.04	3.45 ± 1.42	2.945	0.004
Stratification of average daily screen exposure time [*n* (%)]			8.100	0.017
<1 h/d	13 (36.11%)	5 (12.20%)		
1–2 h/d	14 (38.89%)	15 (36.59%)		
>2 h/d	9 (25.00%)	21 (51.22%)		
Content of screen viewing [*n* (%)]				
Cartoons	31 (86.11%)	38 (92.68%)	0.323	0.570
Educational and intellectual content	3 (8.33%)	2 (4.88%)	0.023	0.880
Entertainment content	21 (58.33%)	26 (63.41%)	0.208	0.648
Parent-child interaction [*n* (%)]			6.856	0.009
≤3 times	16 (44.44%)	7 (17.07%)		
≤3 times	20 (55.56%)	34 (82.93%)		
Reading books [*n* (%)]	11 (30.56%)	7 (17.07%)	1.945	0.163
Telling stories [*n* (%)]	17 (47.22%)	6 (14.63%)	9.718	0.002
Singing [*n* (%)]	16 (44.44%)	11 (26.83%)	2.612	0.106
Taking outdoors [*n* (%)]	31 (86.11%)	32 (78.05%)	0.838	0.360
Playing together [*n* (%)]	21 (58.33%)	17 (41.46%)	2.183	0.140
Recognizing things [*n* (%)]	22 (61.11%)	19 (46.34%)	1.680	0.195
NCATS score	102.56 ± 8.41	97.51 ± 9.57	2.446	0.017
Childcare stress (score)	22.51 ± 1.12	22.58 ± 0.74	0.313	0.756
Dysfunctional parent-child interaction (score)	22.82 ± 0.73	23.24 ± 0.62	2.742	0.008
Difficult children (score)	21.96 ± 0.41	22.04 ± 1.01	0.432	0.668
Passive coping (score)	22.13 ± 3.11	23.24 ± 2.57	1.717	0.090
Emotional outbursts (score)	24.22 ± 3.46	26.45 ± 3.31	2.879	0.005
Aggressive behavior (score)	15.92 ± 2.17	16.31 ± 1.12	0.972	0.335

### 3.11 External validation ROC

**Figure 4:**
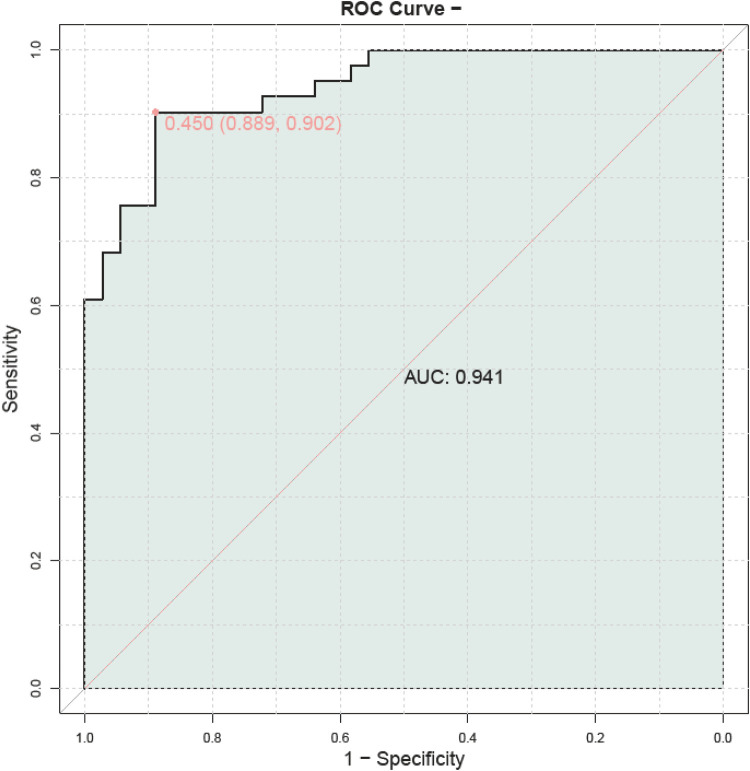
External validation ROC curve.

The provided ROC curve illustrates the diagnostic performance of a model in distinguishing between two classes, with an AUC of 0.941, indicating excellent discriminatory ability (Figure 4). The curve plots sensitivity against 1-specificity at various threshold settings. A point on the curve is highlighted with coordinates (0.450, 0.889), corresponding to a specificity of 0.902, suggesting an optimal balance between true positive and false positive rates at this threshold. The shaded area under the curve visually represents the model's overall performance, demonstrating its capacity to effectively differentiate between the two groups. This high AUC value suggests that the model has strong predictive power for the classification task at hand.

## 4 Discussion

Our study explored how parental screen use habits, young children's screen exposure, and the quality of parent-child interactions collectively influenced the risk of LDD. We found that parents' excessive use of electronic devices not only directly reduced interaction time with their children but also indirectly affected children's language learning opportunities. These factors did not operate in isolation but were interconnected, shaping an environment that either promoted or hindered language development. Through this study, we developed a preliminary risk model that emphasized the importance of evaluating the entire family digital ecosystem rather than focusing solely on the child's individual performance. This approach is consistent with previous research perspectives (24). Our study expands this framework by specifically examining the role of parental screen time and its interaction with other factors, such as parent-child interaction and the child's own screen exposure. By considering these interconnected elements, we can more comprehensively understand how the digital environment shapes language development.

The association between parental ST and language delay warranted particular attention. Our findings support an association between parental screen time and child language development, with similar patterns observed among both mothers and fathers. This adds to the growing body of literature suggesting a link between these factors (25, 26). Even after accounting for children's own screen exposure, this relationship remained, indicating that parents' device use might influence children's language development through multiple pathways beyond merely reducing parent-child interaction time. When caregivers were engrossed in digital devices, their responsiveness to children decreased, leading to fewer spontaneous verbal exchanges (27, 28). These subtle but crucial interactions formed the foundation for early communication skills development. We found that the association between entertainment ST and language development is stronger than that of social media use, suggesting that different types of digital activities have varying impacts on caregiving capabilities. Entertainment content required deeper cognitive engagement, thereby more significantly reducing parents' availability for immediate responses to children.

Our study also highlighted that the amount and content of young children's screen exposure played significant roles in LDD. We found that children in the language developmental delay group had higher average daily screen exposure times compared to those in the normal group. Our findings on ST thresholds aligned with previous research (29). This critical threshold effect held important practical implications, suggesting the need to focus on children's own screen use (30, 31). While educational content might be less harmful, entertainment-focused media often lacked the interactive elements necessary for effective language acquisition. This pattern persisted regardless of content type, challenging the assumption that educational programs could adequately replace interpersonal interactions. Overall, both the duration and type of screen exposure influenced language development outcomes.

Language acquisition required dynamic social reciprocity, joint attention, and real-time feedback, which were difficult to provide through screen viewing (32). Even high-quality educational content could not fully replicate the complex multimodal stimuli that caregivers offered during interactions. In these interactions, caregivers adjusted language input based on the child's responses, providing immediate feedback and support (33).

The quality of parent-child interactions, assessed using the NCATS, revealed another critical aspect of LDD risk. Among various interaction factors, storytelling was identified as being significantly associated with reduced risk of LDD, indicating that it may play a protective role. This finding added a new perspective to research on language enhancement activities, suggesting that certain types of interactions might be especially effective (34). Its unique effectiveness likely stemmed from inherent structural features: narrative sequences naturally incorporated question-and-answer exchanges, contextual reasoning to expand vocabulary, and opportunities for child engagement, all within an emotionally rich context (35). This contrasted with other activities like reading or playing, which could sometimes become routine and lack sufficient language challenges. NCATS scores further emphasized that interaction quality was not just about frequency. Caregivers' responsiveness, such as accurately interpreting children's nonverbal cues, allowing enough time for children to process information, and building on topics initiated by the child, was crucial for language progress (36). When these elements were compromised, whether due to screen-related distractions or other factors, the impact on child development could be significant. Our findings supported existing literature, highlighting the importance of responsive caregiving in establishing communication neural pathways (37).

Emotion regulation strategies were also significantly associated with language development. Emotional outbursts and other difficulties in emotion regulation may disrupt children's learning processes, particularly in language acquisition. Frequent emotional outbursts can make it difficult for children to concentrate, thereby reducing their opportunities to engage in language learning activities. Additionally, emotion regulation issues can affect the quality of parent-child interactions, as children with emotional dysregulation may find it more challenging to communicate and interact effectively with their parents. This further underscores the close connection between emotion regulation abilities and language development (38).

Our study suggests that LDD may result from the combined effects of multiple mechanisms. Parents' excessive use of electronic devices not only reduces interactive time with their children but also decreases their responsiveness, leading to fewer opportunities for spontaneous verbal communication, which is crucial for the development of early communication skills (39). Furthermore, screen exposure to entertainment content has a particularly significant negative impact on language development, likely because it occupies more time and reduces opportunities for high-quality parent-child interactions (7). These factors collectively create an environment that is detrimental to language development, thereby increasing the risk of language delays in children. Understanding these mechanisms can help in developing more targeted interventions to promote children's language development.

Despite our strong findings, the study had several limitations. First, as a single-center retrospective study, the generalizability of our results might have been limited. Recruitment through hospital health checks could bias toward families who frequently engaged with medical services, further limiting the generalizability. The family income and education levels of the participants' parents are generally high, which differs from the regional average. Therefore, our research findings may be more applicable to families with certain socioeconomic backgrounds rather than the entire community. Participants were primarily recruited from specific medical institutions, indicating that they might have higher health awareness and better access to medical resources. This could limit the external validity of the research findings, i.e., their applicability to other regions. The time window of this study overlaps with the COVID-19 pandemic, which may have influenced recruitment practices and the generalizability of the data. Although we made every effort to control for these variables, the economic uncertainty and remote work patterns brought about by the pandemic may still have had some impact on the results.

Additionally, gender, age, and family income have long been considered potential factors influencing children's language development (40). However, in this study, no statistically significant associations were observed between these variables and language developmental delay, which may be attributed to specific cohort characteristics or the relatively small sample size. Additionally, although we used validated tools, ST data relied on parental self-reports rather than objective device tracking, which introduced potential recall or social desirability biases. Furthermore, observations using the NCATS took place in a clinical setting rather than a natural home environment, possibly affecting the authenticity of interactions. This study did not use Variance Inflation Factor to assess multicollinearity, which could result in unidentified multicollinearity among certain variables, thereby affecting the accuracy of regression coefficients. Due to the limited sample size and high correlations among predictor variables, multiple testing corrections were not implemented in this study, which could lead to false-positive significant results. Although we employed various standard methods to evaluate model fit, these methods may not fully capture potential issues in some cases. Therefore, due to data limitations, we were unable to directly test the potential mediating role of parent-child interaction quality in the relationship between parents' screen time and children's screen time. This will be an important direction for future research. Secondly, this study did not control for several potential confounding factors, such as socioeconomic status (SES), home literacy environment, and child temperament, which may affect the interpretation of the results. Additionally, we were unable to rule out the possibility of reverse causality, where delayed language development might lead to increased screen time exposure. Finally, although our model performed well on the validation set (AUC = 0.941), given the small size of the validation set, there is a potential risk of overfitting. The optimal thresholds for “parent-child interaction” and “storytelling” resulted in a sensitivity of 1.0 and a specificity of 0.0. Such cutoff points ensure that no children with delayed language development are missed (i.e., no false negatives), but they also lead to a high number of false positives. This means that many children who are actually developing normally might be incorrectly identified as being at risk, potentially causing unnecessary concern and additional medical examinations.

Future research needed to address these limitations. For example, replicating our findings in larger, multi-center studies could enhance external validity. Prospective longitudinal studies using objective measures could more accurately assess digital behaviors and their impact on child development. Moreover, future research should delve deeper into the specific mechanisms underlying the relationship between ST and language development. Neuroimaging studies could help us understand how excessive screen exposure and disrupted parent-child interactions affect specific neural pathways. Future studies should consider increasing the sample size and diversifying the study subjects to more accurately assess the impact of these factors. Future research should consider broader sampling strategies to ensure that the samples are more representative and can reflect the language development of children from diverse socioeconomic backgrounds.

In conclusion, our study provided valuable insights into how parental ST habits, children's screen exposure, and the quality of parent-child interactions collectively influenced the risk of LDD. By emphasizing the importance of these modifiable environmental factors, our findings highlighted the necessity for early identification and intervention strategies. Future research needed to address the limitations of our study and expand the scope to include a broader range of populations and longer-term outcomes. Ultimately, these efforts would help improve language development in young children and provide better support for families.

## Data Availability

The raw data supporting the conclusions of this article will be made available by the authors, without undue reservation.
